# Variation in Release Factor Abundance Is Not Needed to Explain Trends in Bacterial Stop Codon Usage

**DOI:** 10.1093/molbev/msab326

**Published:** 2021-11-09

**Authors:** Alexander T Ho, Laurence D Hurst

**Affiliations:** Milner Centre for Evolution, University of Bath, Bath, United Kingdom

**Keywords:** release factor, stop codons, translation termination, molecular evolution

## Abstract

In bacteria stop codons are recognized by one of two class I release factors (RF1) recognizing TAG, RF2 recognizing TGA, and TAA being recognized by both. Variation across bacteria in the relative abundance of RF1 and RF2 is thus hypothesized to select for different TGA/TAG usage. This has been supported by correlations between TAG:TGA ratios and RF1:RF2 ratios across multiple bacterial species, potentially also explaining why TAG usage is approximately constant despite extensive variation in GC content. It is, however, possible that stop codon trends are determined by other forces and that RF ratios adapt to stop codon usage, rather than vice versa. Here, we determine which direction of the causal arrow is the more parsimonious. Our results support the notion that RF1/RF2 ratios become adapted to stop codon usage as the same trends, notably the anomalous TAG behavior, are seen in contexts where RF1:RF2 ratios cannot be, or are unlikely to be, causative, that is, at 3′untranslated sites never used for translation termination, in intragenomic analyses, and across archaeal species (that possess only one RF1). We conclude that specifics of RF biology are unlikely to fully explain TGA/TAG relative usage. We discuss why the causal relationships for the evolution of synonymous stop codon usage might be different from those affecting synonymous sense codon usage, noting that transitions between TGA and TAG require two-point mutations one of which is likely to be deleterious.

## Introduction

Most amino acids are encoded by more than one sense codon (Plotkin and Kudla 2011). The three stop codons (TAA, TGA, and TAG) are similarly synonymous in function. In outline, therefore, the well-explored evolution of synonymous sense codons and the less well-explored evolution of stop codon usage might be seen as two sides of the same coin. Being involved in translation termination rather than polypeptide chain extension (Rodnina 2018), however, stop codons do not share molecular machinery with their coding counterparts. Rather than becoming bound to a cognate tRNA (with an accompanied amino acid), the stop codons are instead recognized first by a class I release factor (RF) (Jackson et al. 2012; Rodnina 2018). The resulting complex then becomes bound by a class II RF that mediates peptide release and ribosomal dissociation signaling the end of translation (Jackson et al. 2012; Rodnina 2018).

The precise details of the nature of class I release factors differ between taxa with potential relevance for trends in stop codon usage. In bacteria, TAG is decoded by class I release factor RF1, TGA by class I release factor RF2, and TAA by both RF1 and RF2 (Rodnina 2018). It is most likely that RF1 and RF2 arose via a duplication present in the common ancestor of all bacteria (Burroughs and Aravind 2019). The other domains of life possess one universal RF (Frolova et al. 1994; Inagaki and Doolittle 2000; Jackson et al. 2012; Kobayashi et al. 2012). While the archaeal RF (aRF1) and the eukaryotic one (eRF1) have a very similar catalytic mechanism to the bacterial RFs, the archaeo-eukaryotic lineage RFs appear to be evolutionarily unrelated to bacterial RF1 and RF2 (Inagaki and Doolittle 2000; Vestergaard et al. 2001; Burroughs and Aravind 2019).

Across bacterial species, the usage of the three stop codons varies considerably (Povolotskaya et al. 2012; Korkmaz et al. 2014; Belinky et al. 2018; Ho and Hurst 2019). This variation is not parsimoniously explained by simple covariation with GC content. While TAA usage is negatively correlated with GC and TGA usage is strongly positively correlated, TAG usage, despite having an identical nucleotide content to TGA, is mostly low and unresponsive to GC pressure (Povolotskaya et al. 2012; Korkmaz et al. 2014; Ho and Hurst 2019). In all bacterial species, on average about 20% of all stops is TAG no matter what their GC content (assayed as mean GC3 or GC of the whole genome). As the read-through rate of TAG is consistently lower than that of TGA across species (Strigini and Brickman 1973; Geller and Rich 1980; Parker 1989; Jorgensen et al. 1993; Meng et al. 1995; Sanchez et al. 1998; Tate et al. 1999; Wei et al. 2016; Cridge et al. 2018), the avoidance of TAG, aligned with evidence for the selective importance of read-through avoidance (Bossi and Roth 1980; Wei and Xia 2017; Cridge et al. 2018; Li and Zhang 2019) renders TAG avoidance especially enigmatic. It is suggested that variation in RF1/RF2 cellular abundance may explain this and other features of stop codon usage (Sharp and Bulmer 1988; Korkmaz et al. 2014; Wei et al. 2016).

Sharp and Bulmer (1988) presented one of the earliest RF-based hypotheses to explain bacterial stop codon usage noticing 1) that TAA is the most common stop codon in bacteria and 2) that TAA stop codons can be decoded by both RF1 and RF2 class I release factors while TGA and TAG can only bind one. We now also know that TAA is the preferred stop codon in highly expressed bacterial genes (Korkmaz et al. 2014) consistent with this model. It has additionally been postulated by Wei et al. (2016), by extension of the codon-anticodon adaptation hypothesis (Ikemura 1981, 1992; Akashi and Eyre-Walker 1998; Xia 1998; van Weringh et al. 2011; Prabhakaran et al. 2014; Chithambaram et al. 2014a, 2014b), that bacterial species adjust their stop codon usage to their relative expression of RF1 and RF2, a model we dub the “release factor (RF) hypothesis”. Not only would this explain the apparent preference for TAA, but also potentially the difference in the behavior of TGA and TAG usage against GC content (Povolotskaya et al. 2012; Korkmaz et al. 2014; Wei et al. 2016; Ho and Hurst 2019).

While the RF hypothesis provides an attractive model for explaining TAA optimality in bacteria, both prokaryotic and eukaryotic evidence suggests it is not necessary. While the highly expressed genes of bacteria preferentially use TAA stops (Korkmaz et al. 2014; Wei et al. 2016), so do those of humans (Trotta 2016) where all three stops are decoded by a universal RF. Recently, we also showed that this effect probably is not unique to humans but common across eukaryotes as, controlling for local GC content, TAA usage positively correlates with effective population size (N_e_) (Ho and Hurst 2021). The strongest current explanation for universal TAA optimality is selection for reduced translational read-through (TR), the failure to terminate translation. Indeed, experimentally derived TR rates in both prokaryotes and eukaryotes demonstrate TAA to be the least “leaky” (followed by TAG, with TGA being the most prone to TR) (Strigini and Brickman 1973; Geller and Rich 1980; Parker 1989; Jorgensen et al. 1993; Meng et al. 1995; Sanchez et al. 1998; Tate et al. 1999; Wei et al. 2016; Cridge et al. 2018). We can be confident that TAA is under selection for this purpose due to the similar enrichment of TR-modulating 3′ flanking motifs that reduce TR rates (Bossi and Roth 1980; Wei and Xia 2017; Cridge et al. 2018). At the very least, TR provides a significant selection pressure for TAA regardless of the cellular RF environment.

Nevertheless, the RF hypothesis does receive support from correlations between RF1:RF2 ratios (assessed at mRNA level by real-time qPCR in *Escherichiacoli*, *Mycobacteriumsmegmatis*, and *Bacillussubtilis* and protein level by Western blot in *E. coli*) and TAG:TGA relative stop codon usage observed in a few different bacteria (Korkmaz et al. 2014). It has been noted, however, that analysis of mRNA levels is less informative than protein abundance data as RF2 is translationally regulated (Craigen et al. 1985; Donly et al. 1990; Wei et al. 2016). Subsequent assessment of the RF1:RF2 and TAG:TGA correlation using protein abundance data by Wei et al. (2016) nonetheless corroborated the Korkmaz et al. (2014) result in a wider range of species spanning Proteobacteria, Firmicutes, Cyanobacteria, Actinobacteria, and Spirochetes (*n* = 14). Crucially, Wei et al. (2016) also identify that RF2 is exceptionally low when A + T content at codon third sites (AT3) is high across species. While the RF1:RF2 ratio provides a rationale to explain why TAG and TGA behave differently, that RF2 abundance covaries with AT3 could explain why this difference may vary with GC pressure. With regards to between-species TAG usage trends across GC contents, the authors speculate that at low GC contents mutation bias favors TAA (the most AT-rich stop codon) over TGA and TAG, at mid-range GC contents TAA is favored by selection while TGA is preferred over TAG as RF2 levels exceed RF1, and at high GC contents RF2 is exceptionally high which favors TGA and keeps TAG at low frequency (Wei et al. 2016). The RF hypothesis can hence theoretically explain both the preference for TAA stops (even if it is not the only hypothesis) and the unusual biology of TAG.

Key to understanding the necessity of the RF hypothesis is to solve the TGA/TAG problem. While the above evidence is consistent with the notion that stop codon usage adapts to RF1/RF2 relative levels the causal arrow could predominantly be in the opposite direction: RF1/RF2 usage may adapt to stop codon usage rather than vice versa.

One approach to support the notion that RF1:RF2 ratios adapt to stop usage trends would be to resolve the causes of the anomalous behavior of TAG with respect to GC content. If, for example, we could demonstrate some process that explains invariant TAG usage across genomes that differ widely in GC content then that would lend considerable strength to the notion that another force acts on TAG usage and RF1:RF2 ratios instead respond to equilibrium TAG/TGA ratios. This method is currently problematic. Previously it has been assumed that GC content at putatively neutral sites in a genome must indicate the mutational bias in that genome (Knight et al. 2001). If true, then the neutral AT%/neutral GC% ratio should predict f(TAA)/f(TGA) or f(TAA)/f(TAG) with a line of slope 1. Deviation from this line could then be employed to infer a fixation bias. While this assumes no complex *k*-mer dependent mutation biases (e.g. CpG hypermutability), by far the greater difficulty is that analyses of rare SNPs (Hershberg and Petrov 2010; Hildebrand et al. 2010), mutation accumulation lines (Long et al. 2018), and parent-offspring trios (Smith et al. 2018) report that mutation appears to be universally GC->AT biased. As a consequence, nucleotide content at putatively neutral sites exceeds mutational equilibrium in GC-rich genomes and in GC-rich domains within genomes (Smith et al. 2018). For the same reason, we consider the force causing high GC content to be “GC pressure”, but leave unresolved exactly what the force is, beyond knowing that it is not mutation bias. No matter what the cause, we cannot infer the role of selection by inferring differences between nucleotide usages at a focal site (the termination codon in our case) and some putatively “neutral” site.

An alternative approach that side steps these problems, and the one taken here, is to ask whether the same anomalous trend in TAG usage as a function of GC content is seen when the RF1:RF2 release factor hypothesis does not apply. Here, we focus on the discrepancy between TGA and TAG usage as a function of GC content. We consider several tests. First, if RF1 and RF2 abundance were to explain between-species stop codon usage trends in bacteria then one would not expect to see the same trends in stop codon trinucleotides outside of the canonical termination context. To consider this, we extend our prior analysis (Ho and Hurst 2019) and examine trends in 3′ UTR including after the first downstream stop, allowing for the possibility that the first 3′ UTR stop codon may be a fail-safe codon (Major et al. 2002; Liang et al. 2005; Adachi and Cavalcanti 2009). Second, assuming that termination control should be approximately the same for all genes within any given genome, any covariance between stop codon usage and GC content observed between bacteria genomes should not be repeated in intra-genomic analysis. Third, we take advantage of the fact that the other domains of life possess one universal RF (Frolova et al. 1994; Inagaki and Doolittle 2000; Jackson et al. 2012; Kobayashi et al. 2012) and ask whether the trends seen across bacteria are seen across these groups, most notably Archaea these being close relatives of bacteria. If they are, this would lend weight to the hypothesis that stop codon usage trends do not require an RF1/RF2 mediated rationale.

Given that TGA and TAG have identical nucleotide contents, an anomalous trend in TAG usage has two possible diagnostics. We could consider evidence of a significant positive relationship between TGA usage and GC content in combination with no significant correlation between TAG usage and GC content as one diagnostic. The finding of no significant correlation for TAG versus GC is, however, potentially sensitive to sampling and sample size. We thus also consider as a more general alternative a difference in slope between TAG and TGA usage as a function of GC, with the slope being steeper for TGA.

We test the above predictions using in silico analysis of bacterial, eukaryotic, and archaeal whole-genome sequences. In agreement with our recent studies (Ho and Hurst 2019, 2021), we find stop codon usage to be highly consistent at the canonical stop site and at genomic loci not involved in translation termination. We too find that stop codon usage trends are consistent between bacteria and archaea. Intra-genomically, stop codon usage can be predicted by local (genic) 3′ UTR GC content within the genomes of most bacteria and in humans with TAG anomalous in both. These results are largely consistent with the hypothesis that there are (unspecified) forces external to RF abundance that dictate genome-wide stop codon usage in bacteria and elsewhere. Evoking Occam’s razor, we propose that in bacteria, it is more likely that RF expression adapts to stop codon usage rather than vice versa. We suggest that the evolution of synonymous stop codons and the evolution of synonymous sense codons may well operate according to different principles, the possible reasons for which we discuss. We also provide additional data indicating that TAA optimality appears to be universal regardless of RF diversity.

## Results

### Between-Species Stop Codon Usage Trends in Bacteria are Consistent Outside of the Canonical Termination Site

Our first test of the RF hypothesis concerns stopping codon usage inside and outside of the canonical termination context. As TAA, TGA, and TAG trinucleotides do not function in translation termination in untranslated sequence, the RF hypothesis poses that there is no reason why their usage (and cross-species trends) should reflect what is seen at the canonical stop codon site. Here, then, we consider the relative usage of trinucleotides TAA, TGA, and TAG at the canonical stop site and the 3′ UTRs across bacteria. Note that for this analysis, we use 3′ UTR GC content as our proxy for GC pressure to mitigate the confounding impacts of expression level and codon usage bias. We start by considering whether stop codon usage trends at the focal termination site are the same as those in 3′ UTR. Previously we found that stop codon usage is consistent between the canonical stop site and the 3′ UTR across bacteria (Ho and Hurst 2019). However, as 3′ additional stop codons (ASCs) have been proposed as a potential fail-safe mechanism to prevent phenotypic error (Major et al. 2002; Liang et al. 2005; Adachi and Cavalcanti 2009) the first in-frame “stop codon” downstream of the canonical stop might be subjected to similar termination selection pressures (possibly mediated by RF1:RF2 ratios). To control for this, we expand our analysis to consider only in-frame “codons” downstream of the first occurring in frame ASC.

**Fig. 1.**
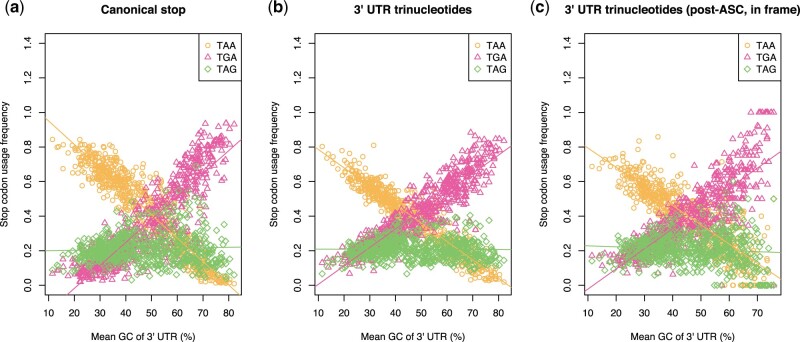
Stop codon usage at (*a*) the canonical stop codon site, (*b*) any 3′ UTR site irrespective of frame, and (*c*) in-frame positions downstream of an ASCas a function of 3′ UTR GC content across 644 phylogenetically independent bacteria. TGA is positively correlated with GC content at all three sites (Spearman’s rank: all *P* < 2.2 × 10^−16^, rho = 0.89 at the canonical stop, rho = 0.93 in the 3′ UTR, rho = 0.86 downstream of ASCs), TAA is negatively correlated with GC content at all three sites (Spearman’s rank: all *P* < 2.2 × 10^−16^, rho = −0.93 at the canonical stop, rho = −0.95 in the 3′ UTR, rho = −0.87 downstream of ASCs), and TAG is unresponsive to GC pressure at all three sites (Spearman’s rank: *P* = 0.79, rho = −0.010 at the canonical stop, *P* = 0.42, rho = −0.032 in the 3′ UTR, *P* = 0.059, rho = −0.074 downstream of ASCs).

We find TGA is positively correlated with GC content at all three sites (fig. 1; Spearman’s rank: all *P* < 2.2 × 10^−16^, rho = 0.89 at the canonical stop, rho = 0.93 in the 3′ UTR, rho = 0.86 downstream of ASCs), TAA is negatively correlated with GC content at all three sites (Spearman’s rank: all *P* < 2.2 × 10^−16^, rho = −0.93 at the canonical stop, rho = −0.95 in the 3′ UTR, rho = −0.87 downstream of ASCs), and TAG is unresponsive to GC pressure at all three sites (Spearman’s rank: *P* = 0.79, rho = −0.010 at the canonical stop, *P* = 0.42, rho = −0.032 in the 3′ UTR, *P* = 0.059, rho = −0.074 downstream of ASCs). Notably, TAG usage is decoupled from TGA usage in 3′ UTR sequences in the same way as at the canonical stop site. TGA and TAG usage slopes against 3′ UTR GC content are significantly different from each other when considering the canonical stop (TGA: 0.013, TAG: 0.00028), 3′ UTR trinucleotides (TGA: 0.011, TAG: −1.1 × 10^−5^), and 3′ UTR in-frame codons downstream of the first occurring ASC (TGA: 0.012, TAG: −0.00056). These results, and prior similar results for out-of-frame “stop” codons in 3’ UTR (Ho and Hurst 2019), suggest that RF1:RF2 dynamics are not required to explain differential trends seen for TGA and TAG.

### The Relationships between Stop Codon Usage and GC Content Observed Between-Species Are Also Observed in Within-Genome Analysis

The RF hypothesis predicts variation in stop codon usage between species possessing different RF1:RF2 profiles. It conversely predicts that no such variation should exist between genes subject to equal RF1 and RF2 availability. As genes within the same genome should be exposed to approximately the same RF environment within any given cell, the hypothesis that stop codon usage is dictated by forces other than RF1:RF2 ratios predicts that GC content and stop codon usage should be intra-genomically correlated. A key question then is whether intra-genomically TGA and TAG respond differently to local GC pressure.

To test for a relationship between stop codon usage and GC content of the same gene, logistic regression was used to predict stop codon usage (as a binary variable where 1 = present and 0 = absent for TAA, TGA, and TAG stop codons of a particular gene) using GC content calculated from the 3′ UTR (Source data, available: https://github.com/ath32/RF). Given the highly noisy nature of the GC content of 3′ UTR (compared to mean UTR GC across all genes in a genome) and the fact that each gene presents only a single stop codon, this analysis is likely to be noisy and underpowered.

We find that TAA usage is well predicted by, and negatively associated with, 3′ UTR GC content across our sample of 644 bacteria. In 640/644 species, there is a negative coefficient when predicting TAA usage with 3′ UTR GC, significantly more than expected by null chance (Binomial test with null *P* = 0.5, *P* < 2.2 × 10^−16^). In 624/644 species, the predictive nature of 3′ UTR GC on TAA usage is both negative and significant with *P* < 0.05, this also far exceeding chance (Binomial test with null *P* = 0.05, *P* < 2.2 × 10^−16^). As predicted by GC pressure alone, we find the opposite trends for both TGA and TAG usage. 3′ UTR GC content is a positive predictor of TGA usage in 622/644 species and of TAG usage in 545/644 species, both ratios being more than expected by chance (Binomial tests with null *P* = 0.5, both *P* < 2.2 × 10^−16^). This positive predictive power is significant in 540/644 species with respect to TGA usage and 413/644 species with respect to TAG usage, more than chance in both cases (Binomial tests with null *P* = 0.05, both *P* < 2.2 × 10^−16^).

That we see evidence for covariance with GC content for both TGA and TAG could be considered consistent with predictions of the RF hypothesis—when RF variation is removed TGA usage and TAG usage respond similarly with GC content. We, however, find evidence of an intragenomic disconnect between TGA and TAG. The estimated coefficient in models predicting TGA usage using 3′ UTR GC content is higher in absolute terms than equivalent models that predict TAG usage in 373/644 cases, this being more than expected by chance (Binomial test with null ratio = 0.5, *P* = 6.7 × 10^−5^). This implies TGA usage is more strongly coupled with local GC pressure than TAG usage, hence the factors underling the between-species TGA/TAG difference could also be present within genomes where RF environment is approximately the same for all genes.

There, however, exists the possibility that for most bacterial genomes that GC content is approximately the same for all genes and hence the signals described above are mostly noise. To mitigate this, we analyze a published subset of bacteria defined as having unusually high intragenomic GC content variation (Daubin and Perriere 2003). We find the same trends are robustly supported (Supplementary table 1, Supplementary Material online). TAA is significantly predicted by genic GC3 in 10/18 genomes, GC3 being a negative predictor in all 10 cases (Binomial test with null ratio = 0.5, *P* = 0.0020). TGA is significantly predicted by genic GC3 in 9/18 genomes, GC3 being a positive predictor in 8/9 cases (Binomial test with null ratio = 0.5, *P* = 0.039). TAG is significantly predicted by genic GC3 in 10/18 genomes, with GC3 a positive predictor in 5/10 cases but a negative predictor in the other 5. For the great majority of genomes within this sample (14/18, 77.8%), the TGA versus GC slope is also more positive than the TAG versus GC slope, consistent with the between-species analysis.

There too exists the possibility that RF1 and RF2 abundance varies throughout the cell cycle. To control for this, one might consider intragenomic analysis of a genome that possesses both substantial GC content variation and only one release factor for translation termination. We hence consider the human genome which possesses both traits due to its isochore structure (Eyre-Walker and Hurst 2001; Galtier et al. 2001; Duret and Galtier 2009) and one universal class I release factor (eRF1). Alongside 3′ UTR GC content, in the human genome, we may too consider coding sequence GC3 content given the relative lack of strong synonymous codon usage bias (Vogel et al. 2010; Plotkin and Kudla 2011). We find that not only can intragenomic stop codon usage be predicted by GC pressure in most bacteria; there too exists a relationship between stop codon usage and GC content in the human genome.

**Fig. 2.**
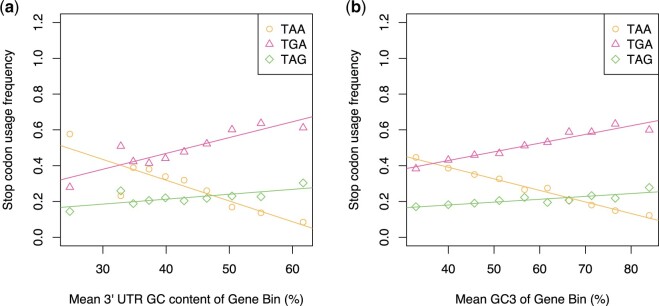
Stop codon usage as a function of (*a*) 3′ UTR GC content and (*b*) GC3 content across the human genome. TGA usage is positively correlated with both 3′ UTR GC content (Spearman’s rank: *P* = 0.0035, rho = 0.85) and GC3 content (Spearman’s rank: *P* < 2.2 × 10^−16^, rho = 0.98). TAA usage is negatively correlated with both 3′UTR GC content (Spearman’s rank: *P* = 0.0068, rho = −0.82) and GC3 content (Spearman’s rank: *P* < 2.2 × 10^−16^, rho = -0.99). TAG usage is not significantly correlated with 3′ UTR GC content (Spearman’s rank: *P* = 0.080, rho = 0.59) but is positively correlated with GC3 content (Spearman’s rank: *P* = 0.0020, rho = 0.88).

When genes are grouped by their 3′ UTR GC content into 10 equal bins (fig. 2*a*), we find TGA usage is positively correlated with 3′ UTR GC content (Spearman’s rank: *P* = 0.0035, rho = 0.85) and TAA usage is negatively correlated with 3′ UTR GC content (Spearman’s rank: *P* = 0.0068, rho = −0.82). TAG usage does not significantly correlate with 3′ UTR GC content (Spearman’s rank: *P* = 0.080, rho = 0.59), in agreement with the disconnect observed between TGA and TAG usage between bacterial species. Results are slightly different when we employ GC3 as the measure of local GC content (fig. 2*b*). Here, we find TGA usage is positively correlated with GC3 content (Spearman’s rank: *P* < 2.2 × 10^−16^, rho = 0.98), TAA usage is negatively correlated with GC3 content (Spearman’s rank: *P* < 2.2 × 10^−16^, rho = −0.99), and, unlike the 3′ UTR GC measure, TAG usage is positively correlated with GC3 content (Spearman’s rank: *P* = 0.0020, rho = 0.88). However, TAG usage is nonetheless decoupled from TGA usage as indicated by a significantly shallower slope (Z-test on slopes; 0.0016 for TAG, 0.0049 for TGA, *P* = 4.8 × 10^−9^) and lower frequency in absolute terms at all GC3 contents. We conclude that the anomalous behavior of TAG can be seen in the absence of RF1:RF2 ratio variation.

### Despite Their Shared Release Factor Recognition, TGA, and TAG Usage are Decoupled across Archaea

**Fig. 3.**
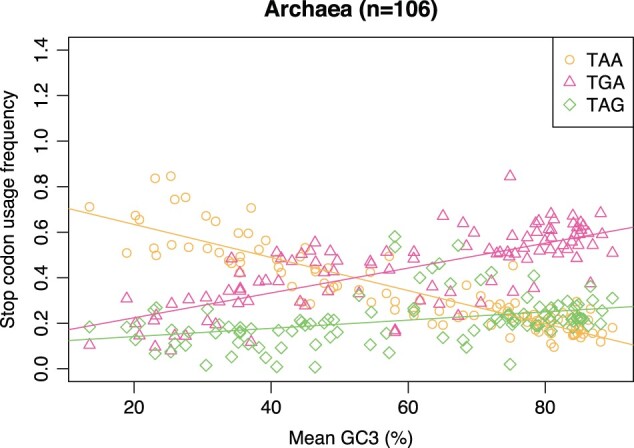
Stop codon usage as a function of 3′ UTR GC content across 106 archaea. TGA usage is positively correlated with 3′ UTR GC content (Spearman’s rank: *P* < 2.2 × 10^−16^, rho = 0.76). TAA usage is negatively correlated with 3′ UTR GC content (Spearman’s rank: *P* < 2.2 × 10^−16^, rho = −0.90). TAG is positively correlated with 3′ UTR GC content (Spearman’s rank: *P* < 2.2 × 10^−16^, rho = 0.57).

Just as the RF hypothesis makes predictions as what to expect in between-species and within-species analysis of bacteria, it too makes predictions regarding the closely related archaea. As archaea use only one class I RF (aRF1) to decode all three stop codons, stop codon usage variation between archaeal species cannot be attributed to RF abundance. If between-species trends were to match the trends seen in bacteria, this would suggest forces external to RF abundance are more significant influencers of stop codon usage. We hence here analyze the stop codon usage of 106 archaeal genomes as a function of 3′ UTR GC content (fig. 3).

We find that, on initial examination, the stop codon usage trends observed in archaea do somewhat resemble what we see in bacteria (fig. 1). TAA and TGA trends are consistent with bacteria observations in archaea, TGA usage being positively correlated with 3′ UTR GC content (Spearman’s rank: *P* < 2.2 x 10^−16^, rho = 0.76) and TAA usage being negatively correlated with 3′ UTR GC content (Spearman’s rank: *P* < 2.2 × 10^−16^, rho = −0.90). TAG usage appears to behave slightly differently between the two domains, however. In bacteria, TAG is unresponsive to GC pressure (fig. 1), while TAG is positively correlated with 3′ UTR GC content across archaea (Spearman’s rank: *P* < 2.2 × 10^−16^, rho = 0.57). Despite this, there is evidence that TGA and TAG usage are differently correlated in archaea, as observed in bacteria. In archaea, the slope of TAG usage against 3′ UTR GC (0.0032) is significantly shallower than the comparable TGA usage trend (0.0079) (Z-test on slopes; *P* = 2.1 × 10^−7^). The RF hypothesis gives no parsimonious explanation for this given that TGA and TAG stop codons are decoded by the same class I release factor in archaea. All these results are repeated when mean GC3 is used as the proxy for GC pressure in the analysis of archaea (supplementary fig. 1, Supplementary Material online).

**Fig. 4.**
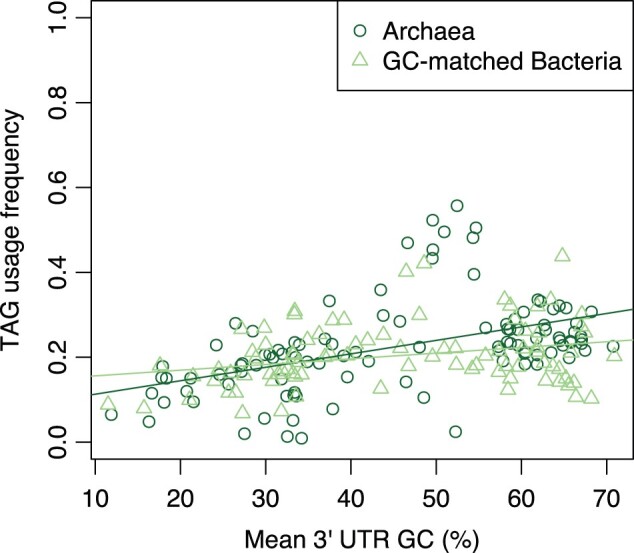
Stop codon usage as a function of 3′ UTR GC content across 106 archaea and GC-matched bacteria. TAG is positively correlated with 3′ UTR GC content in both archaea (Spearman’s rank: *P* < 2.2 × 10^−16^, rho = 0.57) and GC-matched bacteria (Spearman’s rank: *P* = 0.0037, rho = 0.28). The slopes of linear regressions fitted to the two sets of data are significantly different (Z-test: archaea slope = 0.0032, bacteria slope = 0.0013, *P* = 0.0097).

The above analysis, however, is in some regards unfair. Close examination of the bacterial data suggests that at the highest GC levels TAG usage is especially low (fig. 1). The archaeal genomes are, however, not represented in this more extreme end of GC contents. Might the finding of a positive correlation between TAG usage and GC content in archaea but not in bacteria reflect this sampling issue? To assess this possibility, for each archaeal species we select the nearest bacterial species by mean 3′ UTR GC content to be used for comparison between domains. For the two equally sized GC-matched sets of genomes, we then repeat the correlation analysis and test for differences between linear models fitted to TAG usage against 3′ UTR GC content (fig. 4).

In this GC-matched bacterial data set, we find TAG usage to be positively correlated with 3′ UTR GC content (Spearman’s rank: *P* = 0.0037, rho = 0.28), consistent with the archaeal result. Thus, the prima facie modest differences between archaea and bacteria in TAG usage trends appear mostly to be explained by sampling differences. This underscores the notion that TAG’s weak response to GC pressure is independent of RF1:RF2. Note, however, that the slopes of TAG usage against 3′ UTR GC content are still slightly different between the two groups (Z-test: archaea slope = 0.0032, GC-matched bacteria slope = 0.0013, *P* = 0.01).

We conclude that the stop codon usage trends against GC pressure in bacteria and archaea are approximately the same with TAG and TGA being discordant in both. This is not predicted by the RF hypothesis which proposes that the disconnect between TGA and TAG usage in bacteria can be parsimoniously explained by RF1:RF2 ratios without the need to evoke other forces.

We have also analyzed the stop codon usage trends in a sample of eukaryotes, finding TGA and TAG usage to behave near-identically with 3′ UTR GC content in simple linear regression analysis (supplementary fig. 2, Supplementary Material online) and in phylogenetically controlled analysis using PGLS (supplementary table 2, Supplementary Material online). The relevance of this in explaining the bacterial trends is, however, unclear. We see no reason why eukaryotic trends should diminish the archaeal result which supports that the hypothesis that forces external to RF environment is required to explain the TAG/TGA usage disconnect.

### TAA Is the Most Enriched Stop Codon across Bacterial, Eukaryote, and Archaeal Species

Above we have presented several lines of evidence that one need not evoke the RF hypothesis to explain stop codon usage trends in bacteria, most notably the disconnect between TGA and TAG usage (which is observed at the canonical site, in the 3′ UTR, across archaea, in intra-genome bacterial analysis, and in the human genome). We next move away from the TGA/TAG enigma to consider a different prediction of the RF hypothesis: that TAA is optimal in bacteria because it is decoded by both RF1 and RF2 (while TGA and TAG are decoded uniquely by one RF only). As eukaryotes and archaea possess only one class I release factor in their termination machinery, the RF hypothesis predicts TAA to be no more enriched than TGA and TAG. To test this, we calculate stop codon enrichment compared to null expectation for all bacterial, eukaryotic, and archaeal species. The null frequencies in question are produced from 10,000 dinucleotide-controlled simulations (see methods), with enrichment or under-enrichment of stop codon frequency beyond null chance determined by (O-E)/E scores.

Consistent with the RF hypothesis, TAA enrichment exceeds that of TGA and TAG in 74.5% of bacterial species (480/644), this being significantly more than a simple null of 33.3% (one third, as one of the three stops must be the most enriched) (Binomial test; *P* < 2.2 × 10^−16^). We also find TAA to be the most enriched stop in 81.8% (18/22) of our eukaryote genomes (more than expected by chance, *P* = 4.1 × 10^−6^) and 54.7% (58/106) of our archaeal genomes (*P* = 4.9 × 10^−6^), hence forces outside of RF abundance are needed to explain TAA enrichment in such species. This complements evidence that TAA is also preferred in highly expressed genes in numerous taxa (Korkmaz et al. 2014; Trotta 2016; Ho and Hurst 2021).

## Discussion

The RF hypothesis poses that bacterial species match their stop codon usage to their relative expression of RF1 and RF2 (Sharp and Bulmer 1988; Korkmaz et al. 2014; Wei et al. 2016). However, while RF1:RF2 ratios are indeed predictive of stop codon usage trends across bacteria, the causal arrow could be running in the opposite direction. Our data are more supportive of this direction of the causal arrow: between-species trends in stop codon usage are consistent in 3′ UTR trinucleotides that do not function in translation termination, between-species trends in stop codon usage are consistent in within-genome analysis (controlling for RF abundance) in bacteria and in humans, between-species trends in stop codon usage are consistent in archaea which possess only one RF. Furthermore, that TAA is the most enriched stop codon (against dinucleotide-controlled null) across all three domains of life suggests that this phenomenon also cannot be explained by the RF hypothesis. We do not wish to suggest that RF abundance need not play a role in shaping stop codon usage in bacteria. However, it is apparent that other significant forces are needed to explain stop codon usage trends, not least because the same trends are seen in contexts where RF1:RF2 ratios cannot be, or are unlikely to be, causative.

A priori, the notion that RF usage adapts to stop codon usage is possibly more parsimonious from a viewpoint of evolutionary accessibility. If we imagine that RF1:RF2 ratios have, for some reason, shifted, it is not easy to see how TGA<->TAG exchanges might evolve in response to such shifts. To move between these two stop codons, we need a minimum of two mutational events. As the failure to terminate is deleterious (Li and Zhang 2019), we may also assume the intermediate to most often be the third stop codon, TAA, it being one mutation from either TGA or TAG. That two mutations are needed renders such adaptation to the RF pool difficult. That one needs to route via the optimal stop codon renders the process requiring even more special explanation. Imagine, for instance, that selection could favor TGA->TAA because TGA’s release factor is limiting. If so, then TAA->TAG is likely also to be deleterious as the sum of RF1 and RF2 must be greater than the sum of either (and TAA is more generally optimal). By contrast, if one evokes the notion that other forces act on stop codon usage (as our data strongly supports) then adapting the RF1:RF2 pool to better fit such usage is a trivial question of adjusting expression levels of one of either gene. If TGA does not have enough of its release factor then shifting RF1:RF2 levels is a much more evolutionarily accessible solution than two mutational events, one of which is deleterious. On a priori grounds, then adaptation of RF1:RF2 to stop usage seems the more likely mechanism.

We could imagine several experimental tests of this. First, there are strains available with all TAGs replaced with TAA stop codon (Lajoie et al. 2013). Given this, if the RF1:RF2 ratio evolves in response to stop codon usage, as we suggest is more likely, then over several generations we would expect RF1 levels to decrease. Indeed, an RF1 deletion may be favored, much as RF2 is absent from mollicutes that no longer employ TGA as a termination codon (Grosjean et al. 2014). Conversely, we could alter RF1:RF2 ratios by modifying promoters or post-transcriptional regulation. Imagine for example that we could increase RF1 levels leaving RF2 levels static. In principle, TAG should now be favored over TGA under the RF1:RF2 ratio hypothesis. However, we predict that there would be little increase in TAG as one required mutation, TAA->TAG would be just as deleterious as before. The ratio could adjust nonetheless because of an increased substitution rate from TGA to TAA. However, selection always favors TGA->TAA as TAA is optimal. The change in this rate is thus also expected to be marginal. One could argue that deletion of either RF1 or RF2 must cause selection to increase TGA or TAG ratios as one of the two may well be no longer functional as a stop codon. However, this is not an informative experiment as the effect is probably so catastrophic that no organism could tolerate it (or be competitive) unless they already had abolished usage of one of the two G containing stop codons. Thus, the weak selection context involving small changes to RF1:RF2 ratios is by far the more biologically plausible condition.

We note that the hypothesis that RF1:RF2 is adapted to stop codon usage, rather than vice versa as proposed by the RF hypothesis, is not necessarily in disagreement with the observations of Korkmaz et al. (2014) or Wei et al. (2016). The observed correlations between RF1:RF2 and TAG:TGA usage can be explained by either direction of causality or, indeed, by no causality at all and the existence of other covariates. How to explain the disconnect between TAG and TGA usage at high genomic GC content is more difficult. Recalling the model proposed by Wei et al. (2016), the RF hypothesis is not needed to support mutation bias favoring TAA over TGA/TAG at low GC nor selection favoring TAA over TGA/TAG at mid-GC ranges. At high GC contents, however, unknown forces external to RF1:RF2 would likely be needed to explain the large differences observed between TGA and TAG usage.

What then could explain this? The evidence we report above gives several clues. The disconnect between TGA and TAG is 1) common to bacteria and archaea (and possibly some eukaryotes as evidenced by intra-human trends) and 2) unrelated to translation termination given that the between-species stop codon usage trends against GC content are consistent even when we consider untranslated sequences. There are a few candidate hypotheses to explain the enigma of TAG usage. Under-usage of TAG might come as a result of complex mutation biases that disfavor the trinucleotide (or the internal dinucleotides) in all sequences. Alternatively, there could exist purifying selection or other complex fixation biases (GC-biased gene conversion, for example) against such trinucleotides and dinucleotides. The calculation of TGA and TAG mutational equilibrium frequencies across a wide range of bacteria would go some way to resolving possible discriminating mutation biases. Comparisons of these mutational equilibria to TGA and TAG fixed frequencies would similarly give an indication of whether discriminating fixation biases are at play. While we do not have evidence regarding the viability of complex (k-mer contingent) selection or complex biased gene conversion, evidence does support simple (mononucleotide-level) action of both. Possible evidence for selection favoring GC comes from the finding that in nitrogen-fixing bacteria, GC content tends to be higher than in related non-nitrogen fixing bacteria (McEwan et al. 1998), this possibly relating to GC being more nitrogen costly than A and T. More generally, unrelated organisms from the same ecology have similar GC contents (Foerstner et al. 2005). Evidence for biased gene conversion comes from observations of higher GC in recombining genomes (Lassalle et al. 2015) and an association between the presence of the nonhomologous end-joining DNA double-strand break repair pathway and GC content (Weissman et al. 2019).

Aside from explaining TGA and TAG usage differences at high GC content, there is a second possible problem with our interpretation: how to explain the adaptation of sense codon usage bias to tRNA pools. There are clear parallels between the relationships of stop codons with release factors and sense codons with tRNAs, so why should stop codons and release factors coevolve differently with the causal arrow being predominantly in the direction of RF1:RF2 adapting to stop usage rather than vice versa? There are several differences that we think mean that selection on stop codon usage and on sense codon usage might be different.

First, as we discussed above, the problem with stop codon usage is how differential selection for TGA and TAG owing to RF1:RF2 ratios could manifest as selection on mutations altering stop codon usage (two mutations via an optimal intermediary). By contrast, synonymous codons tend to be only one mutational event away from each other. Thus, in the case where codon and tRNAs are out of supply-demand equilibrium (Qian et al. 2012), it is easier to see how a point mutation (unpreferred->optimal codon) can be a viable evolutionarily accessible route.

Second, each gene has only one stop codon but multiple copies of codons for many amino acids. It is hence is easier to see why a tRNA might be translationally rate limiting. Consequently, one can envisage cases when selection favors a tRNA duplication as it provides more of a translational resource for a fast-growing organism (Higgs and Ran 2008). When this happens, supply of that tRNA is likely to exceed demand and so selection could be on codons, especially in highly expressed genes, to use that over-dosed tRNA. By contrast, it is less clear whether, with one-stop codon per gene, selection would prefer more release factor in absolute terms. Indeed it is notable that there appears to be coadaption between tRNA gene copy numbers and amino acid compositions in all three domains of life (McFarlane and Whitehall 2009; Du et al. 2017), while to our knowledge, we do not see similar duplications of *prfA* (encoding RF1) or *prfB* (encoding RF2) across bacteria.

Related to this is the problem of the rate at which RF1 and RF2 can be re-used after being employed in a termination function. In theory, there need be no lag period, while tRNAs must first be amino-acylated. In sum, then, it is not clear that RF1:RF2 levels need to be limiting in the same way tRNA pools might be limiting, meaning selection for RF1 or RF2 duplication that places the system out of supply-demand equilibrium is unlikely to be commonplace. This being said, in vitro experiments suggest different RF concentrations might improve termination, albeit with trade-offs, in certain conditions (Abdalaal et al. 2020) and can affect TR rates (though not translational efficiency per se) (Le Goff et al. 1997).

A final possibility is that sense codon usage and stop codon usage may not be so different and it is codon/stop usage bias that comes first in both cases. However, models presuming that codons and tRNA abundances coadapt, with selection for more tRNA when translation is limiting, provide a good account of data (Shields 1990; Higgs and Ran 2008; Ran and Higgs 2010). This is similarly a wealth of evidence for correlations between tRNA abundance and synonymous codon usage (Varenne et al. 1984; Sorensen et al. 1989; Gingold and Pilpel 2011). We caution, however, that there is no experimental evidence (that we know of) that tRNA abundance change precedes codon usage adaptation. One could imagine a shift in equilibrium introduced by a shift in codon usage, resulting from a myriad of factors, resulting in selection for altered tRNA abundances.

We note also that theoretical parallels cannot be drawn between stop codons and start codons. While ATG is the near-exclusive start codon in eukaryotes and preferred over other NTG start codons in bacteria (Belinky et al. 2017), there does not exist a simple RF1:RF2-like analog and hence there is no RF-like hypothesis to test with regards start codon usage. Indeed, in some species, GTG start codons appear to be poorly transcribed but more readily translated than ATG (Panicker et al. 2015). This suggests a complexity beyond that seen in the selection of stop codon usage.

## Materials and Methods

### General Methods

All data manipulation was performed using bespoke Python 3.6 scripts. Statistical analyses and data visualizations were performed using R 3.3.3. All scripts required for replication of the described analyses can be found at https://github.com/ath32/RF.

### Genome Downloads and Filtering of CDS and 3′ UTR Sequences

A total of 3,727 bacterial genomes were downloaded from the EMBL database (http://www.ebi.ac.uk/genomes/bacteria.html, accessed August 1, 2018), 380 archaeal genomes were downloaded from advanced search of the NCBI assemblies (https://www.ncbi.nlm.nih.gov/assembly/advanced/, accessed July 2, 2021), and 21 eukaryotic genomes were downloaded from Ensembl (https://www.ensembl.org/index.html?redirect=no, accessed July 2, 2021) or Ensembl Protists (https://protists.ensembl.org/index.html, accessed July 2, 2021). For bacterial and archaeal genomes, we filter to retain just one genome per genus to reduce possible bias due to phylogenetic nonindependence. We too filter to retain only genomes over 500,000 base pairs in length to exclude any extremely small (e.g., plasmid) or incomplete genomes. Only genomes decoded by translation table 11 (possessing all three stop codons) were considered for analysis. This leaves a final sample of 644 bacterial, 106 archaeal, and 21 eukaryotic species.

For extraction of coding and 3′ UTR sequences, we obtain the relevant data from the appropriate accompanying GFF files from the same repositories. Coding sequences for all genomes were filtered to retain only those starting with ATG and terminating in TAA, TGA, or TAG. 3′ UTR sequences were obtained in several different ways. Due to a lack of appropriate annotation, for bacteria and archaea we filter genes to retain only those with >30 nts of 3′ intergenic space and assume the 30 nucleotides downstream of the stop codon to be 3′ UTR sequences. For most eukaryotes, we extract coding sequences and exonic sequences and define exonic sequence downstream of the stop codon to be 3′ UTR sequence. This method cannot be used for single-celled eukaryotes downloaded from the Ensembl Protist repository, hence for these, we filter genes to retain only those with >100 nts of 3′ intergenic space and assume the 100 nts downstream of the stop codon to be 3′ UTR.

### Establishing Between-Genome Trends in Stop Codon Usage

Stop codon usage frequencies were calculated at the canonical termination site of all coding sequences for each genome in the bacteria, eukaryote, and archaea data sets. This was repeated at 3′ UTR null sites for comparisons to genomic regions where stop codons do not function in translation termination. Linear models were fitted to stop codon usage frequencies and mean 3′ UTR GC content to determine between-species trends. Trends were tested for correlation using Spearman’s rank tests.

### Intraspecies Logistic Regression Analysis of Bacteria Species

Coding sequences for our bacterial data set were downloaded from Ensembl bacteria (release 51) as described above. For each species, we calculate the 3′ UTR GC content, coding sequence GC3 content, and identify the stop codon used for each gene. We capture this information in CSV files with the stop codon information captured as presence (scored 1) or absence (scored 0) of TAA, TGA, and TAG. The extent to which stop codon usage, as binary variables, may be predicted by GC content in each genome was investigated using logistic regression facilitated by the “glm” function and “family = binomial” parameter in R.

### Intra-Genome Analysis of *H. sapiens*

3′ UTR GC content was calculated for every gene in the *H. sapiens* genome. Genes were subsequently split evenly into 10 bins by their 3′ UTR GC content. In each bin, stop codon usage frequencies at the canonical stop site were calculated. A linear model was then fit to these stop codon usage frequencies and the mean intronic GC contents of the bins to assess the intra-genomic trend. This was repeated using coding sequence GC3 content instead of 3′ UTR GC content. Tests for correlation were facilitated by Spearman’s rank tests.

### PGLS Analysis of Eukaryotes

Phylogenetically controlled regression analyses were completed to test for correlation between 3′ UTR GC content (or GC3 content) and stop codon usage using the PGLS function of the caper package in R (https://CRAN.R-project.org/package=caper). Pagel’s lambda was predicted by maximum likelihood. The phylogenetic trees required for this analysis were generated using TimeTree (Kumar et al. 2017) and are available at https://github.com/ath32/RF in nexus format. Note that this analysis was only used for eukaryotic species where we can be confident in the phylogenetic relationships between species.

### Dinucleotide-Controlled Simulations

Stop codon usage frequencies were compared to expected frequencies generated from 10,000 dinucleotide-controlled simulations. Simulations used Markov models to preserve reading-frame context at dinucleotide resolution as outlined by Ho and Hurst (2019). The first nucleotide of each simulated sequence was selected according to nucleotide frequencies in the coding sequences. Subsequent nucleotides were selected according to dinucleotide frequencies observed in coding sequences until one codon, or three nucleotides, in length. Deviations of the real frequencies compared to null expected frequencies were calculated as:

$$\mathrm{Deviation}=\;\frac{\mathrm{Observed}\;-\;\mathrm{Expected}}{\mathrm{Expected}}.$$

## Supplementary Material

Supplementary data are available at *Molecular Biology and Evolution* online.

## Supplementary Material

msab326_Supplementary_Data

## Data Availability

The data underlying this article are available in the article and in its online supplementary material.
