# Supplementary material for: Variation in Release Factor Abundance Is Not Needed to Explain Trends in Bacterial Stop Codon Usage
Source: Mol Biol Evol. 2021 Nov 9;39(1):msab326. doi: 10.1093/molbev/msab326 (PMC8789281; doi:10.1093/molbev/msab326)
Supplement: msab326_Supplementary_Data [file msab326_supplementary_data.zip › rf_supplement.pdf]

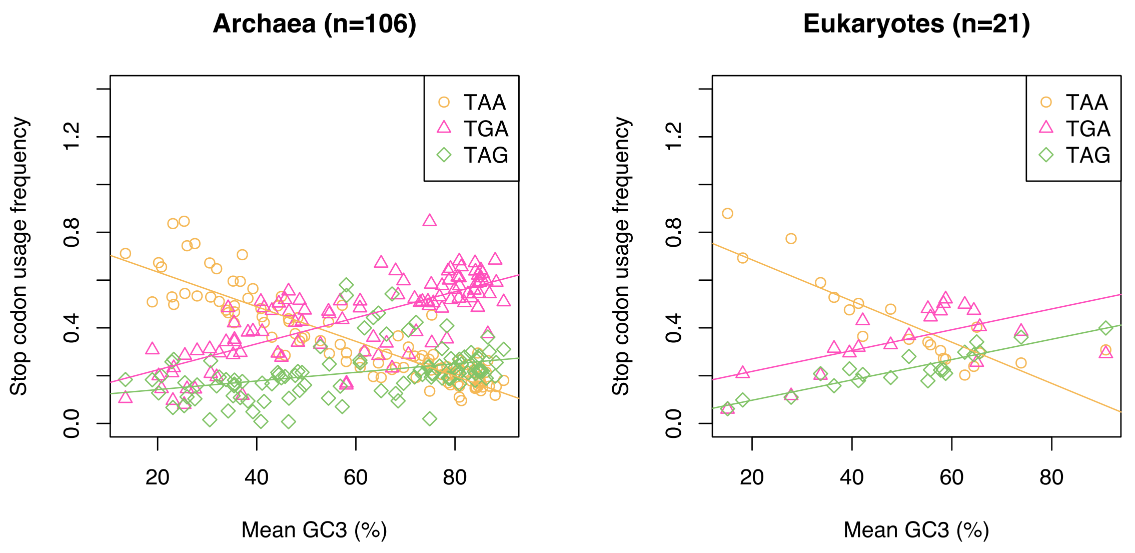

Supplementary figure 1. Stop codon usage as a function of GC3 content across 106 archaea and 21 eukaryotes. TAA usage is negatively correlated with GC3 content across archaea (Spearman's rank: $p < 2.2 \times 10^{-16}$, rho = -0.89) and eukaryotes (Spearman's rank: p = 0.00059, rho = -0.86). TGA usage is positively correlated with GC3 content across archaea (Spearman's rank: $p < 2.2 \times 10^{-16}$, rho = 0.76) and eukaryotes (Spearman's rank: p = 0.012, rho = 0.56). TAG is positively correlated with GC3 content across archaea (Spearman's rank: $p = 1.1 \times 10^{-7}$, rho = 0.49) and eukaryotes (Spearman's rank: $p = 9.5 \times 10^{-7}$, rho = 0.88).

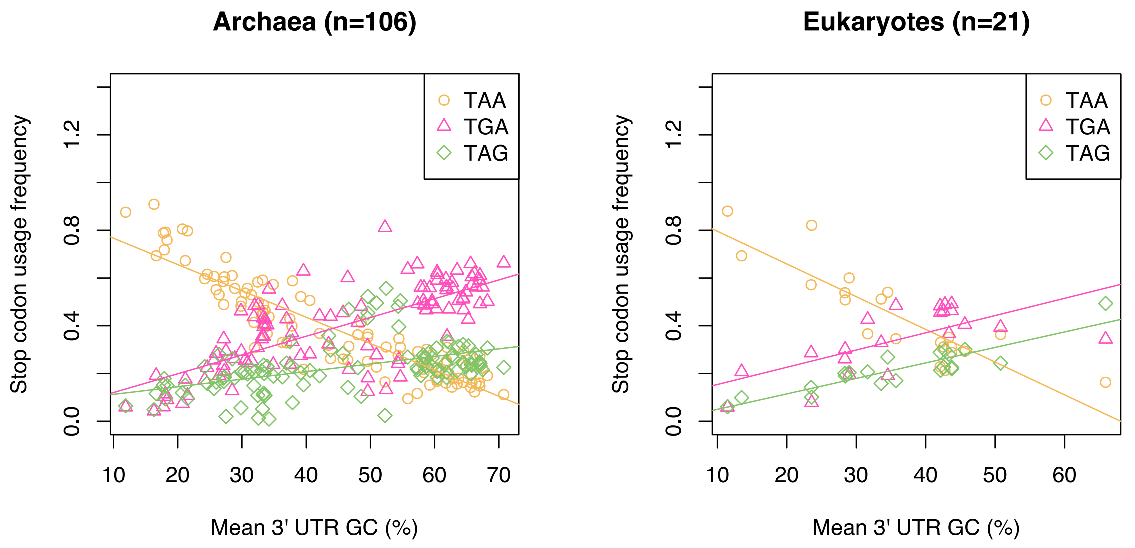

Supplementary figure 2. Stop codon usage as a function of 3' UTR GC content across 106 archaea and 21 eukaryotes. TAA usage is negatively correlated with 3' UTR GC content in archaea (Spearman's rank: $p < 2.2 \times 10^{-16}$, rho = -0.90) and eukaryotes (Spearman's rank: $p < 2.2 \times 10^{-16}$, rho = -0.85). TGA usage is positively correlated with 3' UTR GC content (Spearman's rank: $p < 2.2 \times 10^{-16}$, rho = 0.76) and eukaryotes (Spearman's rank: p = 0.0013, rho = 0.66). TAG is positively correlated with 3' UTR GC content (Spearman's rank: $p < 2.2 \times 10^{-16}$, rho = 0.57) and eukaryotes (Spearman's rank: $p < 2.2 \times 10^{-16}$, rho = 0.85).

Supplementary table 1. Results from logistic regression models predicting intra-genome stop codon usage as a function of GC3 content in 18 bacterial species with unusually high GC3 variance.

Species	TAA		TGA		TAG		TGA slope > TAG slope?	1st Quar. GC3	Median GC3	3rd Quar. GC3
	Coefficient	P-value	Coefficient	P-value	Coefficient	P-value				
Bacillus halodurans	-0.02841	4.74E-07	0.011942	0.0724	0.031814	8.74E-06	-	38.79	42.39	45.61
Bacillus subtilis	-0.005203	0.141961	0.004036	0.31	0.003759	0.441	+	37.84	43.6	48.36
Brucella melitensis	-0.001085	0.8379	0.006046	0.213	-0.009717	0.1398	+	62.9	67.22	70.53
Chlamydia trachomatis	-0.029705	0.000139	0.040822	1.13E-05	0.003748	0.654823	+	30.312	34.032	37.326
Deinococcus radiodurans	-0.0007681	0.8812	0.005232	0.259	-0.010696	0.1175	+	81.33	85.25	87.76
Escherichia coli	-0.0007345	0.80073	0.00871	0.00465	-0.023183	4.70E-06	+	48.5	54.93	59.81
Listeria monocytogenes	-0.047371	2.16E-09	0.038682	3.03E-05	0.03836	0.00036	+	25.35	28.42	31.84
Mycobacterium tuberculosis	-0.02757	0.000165	-0.015345	0.00553	0.037028	3.56E-09	-	76	79.41	82.54
Mycoplasma genitalium	-0.02574	0.112	-3.42E-15	1	0.02574	0.112	-	18.775	22.528	27.414
Mycoplasma pneumoniae	0.007537	0.479	-9.63E-16	1	-0.007537	0.479	+	34.91	41.12	45.81
Pasteurella multocida	0.0003631	0.965	0.003999	0.725	-0.003915	0.706	+	31.25	34.78	37.96
Pseudomonas aeruginosa	-0.052776	<2e-16	0.049092	<2e-16	-0.024599	<2e-16	+	78.74	86.44	89.8
Ralstonia solanacearum	-0.02692	1.73E-07	0.029694	1.71E-11	-0.018197	0.00109	+	82.77	87.6	90.6
Salmonella enterica	0.003003	0.355	0.002948	0.396	-0.014898	0.00401	+	53	59.34	63.56
Sinorhizobium meliloti	-0.020731	1.49E-06	0.019668	1.52E-08	-0.009917	0.0189	+	72.5	77.95	81.45
Staphylococcus aureus	-0.055096	1.89E-09	0.0497	3.57E-05	0.04103	0.000152	+	19.831	22.549	25.424
Thermotoga maritima	-0.035571	5.52E-05	0.033554	2.24E-05	-0.01379	0.2672	+	48.78	52.54	56.21
Vibrio cholerae	-0.010944	0.0283	0.003426	0.563	0.014622	0.0262	-	44.23	48.67	52.3

Supplementary table 2. Results from Phylogenetic Generalized Least Squares (PGLS) analysis testing for correlation between stop codon usage and two proxies of GC pressure. Both genomic 3' UTR GC content and genomic GC3 content are significant predictors of TAA, TGA and TAG usage when controlling for phylogeny. The direction of the correlation of stop codon usage with 3' UTR GC content is indicated by the sign of the estimate. Lambda was computed by maximum likelihood and explains the extent to which the traits are evolving as expected given tree topology alone (0 = each point is phylogenetically independent, 1 = traits are evolving as expected given the phylogenetic relationships).

**Stop codon usage**	**Predictor variable**	**Estimate**	**Adjusted r^2**	**P-value**	**Lambda (ML)**
TAA	Mean GC3	-0.0091	0.80	1.6×10^{-8}	0.83
	Mean 3' UTR GC	-0.014	0.84	5.1×10^{-6}	0.986
TGA	Mean GC3	0.0041	0.37	0.012	0.98
	Mean 3' UTR GC	0.0076	0.56	0.0024	1.000
TAG	Mean GC3	0.0042	0.80	1.1×10^{-5}	0.80
	Mean 3' UTR GC	0.0058	0.66	2.7×10^{-4}	0.000
