# Supplementary material for: Variation in Release Factor Abundance Is Not Needed to Explain Trends in Bacterial Stop Codon Usage
Source: Mol Biol Evol. 2021 Nov 9;39(1):msab326. doi: 10.1093/molbev/msab326 (PMC8789281; doi:10.1093/molbev/msab326)
Supplement: msab326_Supplementary_Data [file response_to_reviewers.pdf › response_to_reviewers.pdf]

Response to reviewers – MBE

Reviewer: 1

Please see reviewer 1's PDF in the submission files to best view their comments

"The paper discusses variation in stop codon frequencies among bacterial genomes and concludes that this is not the result of variation in release factor frequencies, as was argued previously. The paper makes some good arguments, although I have a few questions for clarification.

1. For a genome with GC content p, if TAA and TGA codons were in mutational equilibrium, we would expect the relative frequencies of the codons to be determined by the relative rates of forward and backward mutations

F(TGA)/F(TAA)=p/1-p

If, in addition, there were some negative selective effect against TGA relative to TAA, then we would have

F(TGA)/F(TAA)=p/1-p exp $(- S_{TGA})$

where the exponential factor is equal to the relative rate of fixation of forward and backward mutations. Similarly, for TAG, we have

F(TAG)/F(TAA)=p/1-p exp $(- S_{TAG})$

This suggests that if we plot the ratios f(TGA)/f(TAA) and f(TAG)/f(TAA) against p/(1-p) we will obtain two straight lines with slopes that tell us the selective effects. The fact that f(TGA) responds quickly to changes in p (as in Fig 1) suggests that there may be almost no selection against TGA. Is the data consistent with S_{TGA} = 0? On the other hand, there appears to be definite selection against TAG in Fig 1, so we should find S_{TAG} > 0. If there is systematic variation of selection among the genomes, then these graphs will be curves not straight lines. So it would be interesting to check whether the data is consistent with straight lines.

In contrast, if the codon frequencies are simply plotted against p, as in Fig 1, then we expect $f\,(1 - p)\, p\exp(-S_{TGA})\,(TAA) = (1 - p) + p\exp(-S_{TGA}) + p\exp(-S_{TAG})$, $f(TGA) = (1 - p) + p\exp(-S_{TGA}) + p\exp(-S_{TAG})$, $f(TAG)\, p\exp(-S_{TAG}) = (1 - p) + p\exp(-S_{TGA}) + p\exp(-S_{TAG})$. These graphs are not quite straight lines, which makes it difficult to tell the strengths of selection, and makes the fitting of the data by straight line correlation functions slightly questionable."

Response:
We are very grateful for the thoughtful suggestions from the referee. However, the core premise of these tests is now known to be wrong. The referee assumes that GC content at

putatively neutral sites must be indicative of the mutational bias within each genome. Were this true then p/(1-p) would indeed provide null expectations for f(TGA)/f(TAA) under a mutation bias/neutral null and deviations from this could then be employed to assay fixation bias (which may or may not be selection). This assumption we note was extremely commonplace and indeed, for example, the important paper of Knight et al.[1] showing that GC% is the universal best predictor of codon usage patterns assumed that the genomic GC must tell you about mutation bias. However, multiple subsequent analyses either using low frequency SNPs (bacteria), MA lines (eukaryotes) or parent offspring sequencing (humans, a few eukaryotes), all find the same thing: no matter what the genomic GC (or intronic GC, GC3 etc), mutation is universally GC->AT biased. Thus p/1-p is not a metric of null expectations under a mutation bias null model. Consequently, the above tests do not serve the utility that the referee suggests.

The referee's comment is nonetheless extremely valuable because we realise that other readers may well also wonder why we don't do tests like this. We briefly alluded to the issues in the introduction, noting that one approach to resolve the TAG/TGA enigma is to try and work out, as the referee suggests, what forces are acting. If the referee's method were to work, then it would be wonderful. However, with complex mutation bias, one that is uniformly GC->AT biased, the possible impact of biased gene conversion as well as selection, we found this to be a near impossible task presently. The novelty of our approach is that we found a way to sidestep this approach simply by asking whether we see the same trends when RF1/RF2 ratios could not explain the trends. We have added to the introduction more detail on why the "unpick the causes" approach is currently non-optimal and why in turn, our approach is we think rather an elegant means to show that TGA/TAG enigma cannot be simply owing to RF1:RF2 ratios but leave open what the actual cause might be. We further elaborate on possible causes in the discussion noting both evidence for selection and biased gene conversion favouring high GC against the direction of mutation bias.

1. Knight et al. 2001. Available: doi: 10.1186/gb-2001-2-4-research0010.

2. If selection on stop codon usage were related to speed of action of the release factors, then we would expect the selection coefficients to depend on relative concentrations of the release factors, which is argues not to be the case here. However, there are ways in which selection could depend on the release factors without depending on their concentrations. TAG is decoded by RF1, TGA by RF2 and TAA by both. Suppose that RF2 is much more efficient that RF1, in which case TAA will always be dealt with by RF2, and there will be a selection against TAG only because it is reliant on RF1. But it may not be the speed that matters. Suppose RF1 has a high probability of readthrough. This would lead to a negative selection on TAG that occurs because of the release factors but does not depend on the relative concentrations of the release factors. Readthrough is mentioned in the paper, but perhaps it could be clarified why selection due to readthrough would be different from selection due to speed of release factors.

Response:
The referee we think is making an interesting point that we haven't seen discussed. The observation is that RF1:RF2 ratios do predict TAG/TGA usage but the referee is asking whether, given what we know about stop codon function, this is actually predicted. The referee interestingly suggests that the force may not be rate of processing (which probably

does predict the correlation of ratios) but efficiency at preventing read-through per se. In effect the referee is also asking whether the original model actually works in theoretical terms. We had a theoretical objection (TAG<->TGA takes two mutation one of which must be deleterious as TAA is optimal). The referee raises a second: if TAG simply has high read-through rates, we might expect general selection against TAG to explain its low usage the ratio. We think we can reject the referee's concern as the empirical data says that TGA has the highest read-through rates. We really should have mentioned this as in part this really does underscore the perplexing nature of TAGs rarity and TGA higher usage. We now explicitly mention this in the introduction.

3. Nevertheless, the paper shows that the effects also arise in archaea (where the release factors are different) as well as in the UTR downstream of the stop codon. Therefore the main conclusion of the paper is that the variation in stop codon frequencies in bacteria is not due to release factors at all, and I find this quite convincing. Unfortunately however, the paper is not very clear what the alternative explanation is. To me, the most likely explanation is that there is some context dependent mutational process, so that the GC content is not sufficient to predict the triplet frequencies, and triplets with the same GC content would not necessarily have the same frequency in absence of selection. This could be assessed by measuring relative frequencies of TAA, TGA and TAG in UTRs out of frame of the stop codon. Maybe this has already been done in Figs 1b & 1c? It is not quite clear to me what exactly is included in 1b and 1c, and whether this already makes the comparison of in-frame and out-of-frame triplet frequencies.

Response:
This somewhat reiterates point 1. The tests given here presume – against the evidence – that nucleotide usage in GC rich genomes is indicative of the mutational bias when it is not. This is why we think our sidestep is helpful and original. We did discuss possible explanations for the trends, but we hope that in being more explicit that we can reject mutation bias in GC genomes helps others see that the sort of test presented doesn't arbitrate on the issue. In 1b we consider all 3' stop trinucleotides and in 1c we consider only in frame "stops". We cite an analysis of out-of-frame trinucleotides which finds the same trends. In this case, however, we can't be certain they aren't functional stops to catch frameshift errors.

4. At line 255 there is an analysis of the relationship between stop codon usage and the GC content of the 3'UTR of individual genes. It should firstly be checked whether there is significant variation among genes. Can we reject the hypothesis that the GC content is the same for all genes in one genome? If there is significant variation of GC content among genes in one genome, what causes this variation? If the effect arises from context-dependent mutation, as suggested above, then mutational effects will have to vary systematically in different places in the same genome.

Response:
In bacteria there are well described non-random deviations in GC content (e.g. Ori v Terminus) so a priori we could reject a null on these grounds. It has been presumed that the cause of this is mutational, but we don't think the evidence is unambiguous. The referee makes an important point – what if some genomes aren't like this and we are just analysing so much noise. We were indeed worried that the test might be too conservative because of

such noise (we falsely accept the null because of random variation). We voiced these fears in the text. However, we chose to present an unfiltered data set as to do otherwise might lead the referees (and readers) to suspect that we were P hacking – just searching for significance in a select few genomes. That we find a deviation from null in the complete data set – despite the noisiness of the analysis – we find quite compelling *because* it is an unbiased/unfiltered dataset. We hope the referee appreciates our desire to be as conservative and as unbiased as possible.

However, now that the referee invites us to filter, we are happy that we can do so without accusations of P hacking. We now identify a published sample of genomes defined as having unusually high GC variation. We find the same trends are robustly supported, TAA being negatively correlated with GC, TGA positively correlated, and TAG as often positively correlated as negatively correlated with no significant trend. Similarly in the great majority, the TGA vs. GC slope is steeper than the TAG vs. GC slope as seen in the between-species analysis. This is now added to the manuscript.

5. The paragraph beginning at line 413 discusses which codon is "most enriched". I would suggest changing this to "most frequent". The word enriched seems to imply that the frequency is increased relative to some prior expectation, but there is no prior expectation in this paragraph. I think here what is meant is simply which codon is most frequent.

Response:
We think the referee must have missed the end of the prior paragraph where we define enrichment – and it is enrichment not frequency:

"To test this, we calculate stop codon enrichment compared to null expectation for all bacterial, eukaryotic, and archaeal species. The null frequencies in question are produced from 10,000 dinucleotide-controlled simulations (see methods), with enrichment or under-enrichment of stop codon frequency beyond null chance determined by O-E/E scores."

6. At line 523 it is stated that there is no evidence that tRNA abundance change precedes codon usage adaptation. This is trying to say that tRNAs follow codon frequencies and not the other way. But there are several papers discussing codon frequency evolution that show that there are alternative stable states of codon usage and tRNA gene copy numbers [1-3]. This means the tRNA influence the codons as well. The interaction goes both ways.

[1] Higgs PG, Ran W (2008) Coevolution of codon usage and tRNA genes leads to multiple stable states of biased codon usage. *Mol. Biol. Evol.* 25: 2279-2291.

[2] Ran W, Higgs PG (2010) The influence of anticodon-codon interactions and modified bases on codon usage in bacteria. *Mol. Biol. Evol*. 27: 2129-2140.

[3] Shields DC (1990) Switches in Species-Specific Codon Preferences: The Influence of Mutation Biases. J Mol Evol (1990) 31:71-80

Response:
We are confident that the referee is correct – that change in tRNA copy numbers can drive changes in codon usage. The models presume this and do a pretty good job of explained

the data. The point we were making is that we don't know experimental evidence for this – we should have been more clear. We are very happy to cite the papers below as indeed they strongly support the possibility. We also clarify our point.

Reviewer: 2

The manuscript by Ho and Hurst presented a comprehensive analysis on bacterial release factor (RF) abundance and stop codon usage. The analysed results show that RF1/RF2 ratios become adapted to stop codon usage. The manuscript also discussed why the causal relationships for the evolution of synonymous stop codon usage might be different to those affecting synonymous sense codon usage, noting that transitions between TGA and TAG require two steps one of which is likely to be deleterious. Albeit I am not an expert in this topic, it is very interesting for me to read through the whole manuscript, with the following comments hopefully helpful, yet possibly bizarre, for further improvements.

Response:
We are gratified that the reviewer found the paper very interesting.

1. Different stop codons are under different selection with different read-through rates (TR). For a specific stop codon, is there any difference for bacteria with different GC contents? TR acts more significant selection for TAA. Is this universal for all bacterial species? Does that mean TAA is more ancient stop codon in contrast to TGA and TAG.

Response:
What interesting questions! We don't think we can do more than speculate on which stop might be ancestral, so would prefer not to go there. But the universality of TAA optimality we can comment on. We find this to be remarkably common (75% of our genomes show enrichment for TAA, way more than the 33% null). But is TAA also having low read through rates? This has been measured in a few species, but no link has been made to genomic GC – even via some kind of meta-analysis approach there would not be enough data to say anything meaningful. We can however look at TAA enrichment vs. GC and assess whether TAA might be more preferred in low/high GC species. When we do so, however, we find the degree of TAA enrichment is approximately invariant to GC content suggesting TR rates do not covary with GC and this is not an issue. See the below plot:

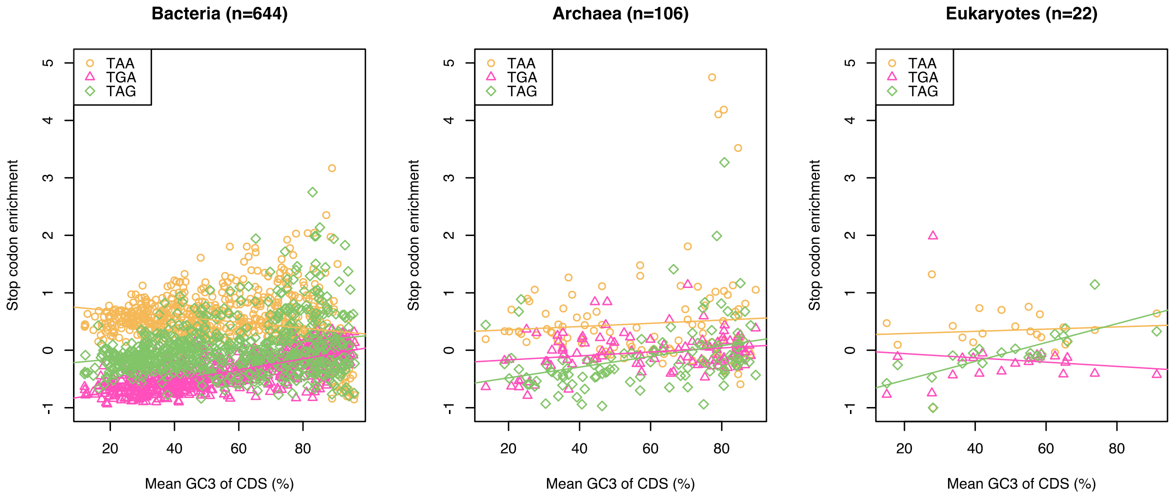

Figure X. Stop codon enrichment above dinucleotide-controlled simulations (O-E/E) for bacterial, archaeal, and eukaryotic species as a function of genomic GC3 content.

2. As reported in the manuscript, TAA is negatively correlated with GC content, TGA is positively correlated, and TAG is insensitive to GC pressure. My understanding is the three codons can play balance roles with GC variation in bacteria. Is that correct?

Response:
Our apologies but we aren't clear what the referee means by balance roles.

I am just curious how about species with other genetic codes (e.g., invertebrate mitochondrial code), which only have two stop codons?

Response:
We did have a look at the translation table 4 bacterial genomes, but they are all very AT rich and considering one genome per genus, very few have been sequenced. There just isn't enough power in the data. We've added mention to the manuscript that those genomes who have lost TGA have also lost RF2, this being important because it says there is a strict link between TGA and RF2.

3. Stop codon usage is under selection for termination. Is this some kind of complement/consistent, in terms of nucleotide content, with start codons that might be also under selection for translation initiation?

Response:
In eukaryotes the start codon is almost exclusively ATG. However, in bacteria we see NTG is the more general rule, albeit with ATG predominating and strongly favoured[1]. However, in this instance there doesn't seem to be a simple RF1:RF2 like analogue, making comparison mute. Moreover, in some species GTG seems to be very poorly transcribed but more efficiently translated than ATG[2]. This suggests a complexity beyond that seen in the stop codon context. We have added comments to this effect at the end of the manuscript.

1. Belinky et al. 2017. https://doi.org/10.1038/s41598-017-12619-6
2. Panicker et al. 2015. https://doi.org/10.1371/journal.pone.0127911

4. Full name is preferred for the first occurrence of AT3 on Line 132.

Response:
AT3 is like GC3: A+T content at codon third sites. Now corrected.

5. Consistent shapes/colors would be better used in all figures.

Response:
We agree and have now updated Figure 2. Figure 4 is slightly different as both sets of points are referring to TAG frequency. We hope the two shades of green here are at least somewhat consistent to the earlier plots.

Reviewer: 3

The authors here attempted to solve a chicken-egg problem; whether RF1/RF2 abundance governs the frequency of UAG/UGA as stop codons or vice versa. This is certainly one of the unresolved questions of bacterial protein synthesis and also important from the evolutionary perspective. The authors extend the analysis of UAA/UAG/UGA abundance in the 3' untranslated sites in bacterial genome, which do not participate in translation termination, and across archaeal species, which use a single release factor instead of two. While I personally agree with their conclusion that RF1/RF2 ratio is unlikely to be the cause for UAG/UGA ratio, I do not think that there work resolves the issue with concrete evidence that the reverse is true. I feel that the work basically demonstrates that UAG is in general the minor stop codon in bacteria and archaea irrespective of its location within or outside the genes and UAG frequency is not related directly to the GC content of the genome (as shown earlier in multiple works cited herein). So, I suggest amendment of the claims in the abstract, introduction and discussion.

Response:
While the referee partially captures out findings, we think that this doesn't fairly reflect the inferences that can be drawn. In particular, if we know that the enigmatic trend difference in TAG and TGA – the stimulus for the RF1: RF2 hypothesis – is also seen in contexts where RF1:RF2 ratio is irrelevant (in bacteria in 3' UTR, in archaea with one RF, across the human genome with one RF) then we haven't just established that TAG is the rare codon but also that the RF1:RF2 is not needed to account for this. The logic of our test and the serious challenge it presents to the RF1:RF2 hypothesis is robust.

Where we agree with the referee is that this line of argument, while strong, is not "concrete", but we feel that we were adequately judicious in our wording:

In the discussion:

"We do not wish to suggest that RF abundance need not play a role in shaping stop codon usage in bacteria. However, it is apparent that other significant forces are needed to explain

stop codon usage trends, not least because the same trends are seen in contexts where RF1:RF2 ratios cannot be, or are unlikely to be, causative."

"The observed correlations between RF1:RF2 and TAG:TGA usage can be explained by either direction of causality or, indeed, by no causality at all and the existence of other covariates."

In the abstract:

"We conclude that specifics of release factor biology are unlikely to fully explain TGA/TAG relative usage."

However, we agree with the referee that the title might be a bit strong. To this end, taking on board the referee's concerns, we think a more appropriate title is:

"Variation in release factor abundance is not needed to explain trends in bacterial stop codon usage"

We hope the referee approves of this change.

I also think that a concrete conclusion can only be reached when this work can be coupled with experimental validation. Although not easy it is doable, which will make the work fitting to the visions and quality of the MBE journal. As we know there are strains available with all UAGs replaced with UAA stop codon (Lajoie MJ, et al., Science. 2013 Oct 18;342(6156):357-60). One can evolve that stain and estimate the relative ratio of RF1 and RF2. If UAG/UGA ratio would govern RF1/RF2 ratio, as it is claimed by in silico analysis in this work, then after evolution of the UAG deleted strain for significant number of generations the fraction of RF1 would presumably decrease. The reverse can also be done, but that will require replacing significant number of UGAs with UAG. However, in my opinion experimental validation would be required to consolidate the claim made by the authors in this paper.

Response:
We agree that experimental follow up to this study would be valuable – but is beyond the scope of the present study. We now suggest a few tests in the discussion in the hope that we might inspire someone to do the experiments. The reviewer's suggestion above provides a lovely test. We also suggest modulation of RF1:RF2 ratios to see if, as we predict no change TGA<->TAG rates.
