# Supplementary material for: Variation in Release Factor Abundance Is Not Needed to Explain Trends in Bacterial Stop Codon Usage
Source: Mol Biol Evol. 2021 Nov 9;39(1):msab326. doi: 10.1093/molbev/msab326 (PMC8789281; doi:10.1093/molbev/msab326)
Supplement: msab326_Supplementary_Data [file Reviewer1_comments.pdf › Reviewer1_comments.pdf]

Ho and Hurst

The paper discusses variation in stop codon frequencies among bacterial genomes, and concludes that this is not the result of variation in release factor frequencies, as was argued previously. The paper makes some good arguments, although I have a few questions for clarification.

1. For a genome with GC content p, if TAA and TGA codons were in mutational equilibrium, we would expect the relative frequencies of the codons to be determined by the relative rates of forward and backward mutations

$$\frac{f(TGA)}{f(TAA)} = \frac{p}{(1-p)}$$

If, in addition, there were some negative selective effect against TGA relative to TAA, then we would have

$$\frac{f(TGA)}{f(TAA)} = \frac{p}{(1-p)}\exp(-S_{TGA})$$

where the exponential factor is equal to the relative rate of fixation of forward and backward mutations. Similarly, for TAG, we have

$$\frac{f(TAG)}{f(TAA)} = \frac{p}{(1-p)}\exp(-S_{TAG})$$

This suggests that if we plot the ratios f(TGA)/f(TAA) and f(TAG)/f(TAA) against p/(1-p) we will obtain two straight lines with slopes that tell us the selective effects. The fact that f(TGA) responds quickly to changes in p (as in Fig 1) suggests that there may be almost no selection against TGA. Is the data consistent with $S_{TGA} = 0$? On the other hand, there appears to be definite selection against TAG in Fig 1, so we should find $S_{TAG} > 0$. If there is systematic variation of selection among the genomes, then these graphs will be curves not straight lines. So it would be interesting to check whether the data is consistent with straight lines.

In contrast, if the codon frequencies are simply plotted against p, as in Fig 1, then we expect $f(TAA) = \frac{(1-p)}{(1-p) + p\exp(-S_{TGA}) + p\exp(-S_{TAG})}$, $f(TGA) = \frac{p\exp(-S_{TGA})}{(1-p) + p\exp(-S_{TGA}) + p\exp(-S_{TAG})}$, $f(TAG) = \frac{p\exp(-S_{TAG})}{(1-p) + p\exp(-S_{TGA}) + p\exp(-S_{TAG})}$. These graphs are not quite straight lines, which makes it difficult to tell the strengths of selection, and makes the fitting of the data by straight line correlation functions slightly questionable.

2. If selection on stop codon usage were related to speed of action of the release factors, then we would expect the selection coefficients to depend on relative concentrations of the release factors, which is argues not to be the case here. However, there are ways in which selection could depend on the release factors without depending on their concentrations. TAG is decoded

by RF1, TGA by RF2 and TAA by both. Suppose that RF2 is much more efficient that RF1, in which case TAA will always be dealt with by RF2, and there will be a selection against TAG only because it is reliant on RF1. But it may not be the speed that matters. Suppose RF1 has a high probability of readthrough. This would lead to a negative selection on TAG that occurs because of the release factors but does not depend on the relative concentrations of the release factors. Readthrough is mentioned in the paper, but perhaps it could be clarified why selection due to readthrough would be different from selection due to speed of release factors.

3. Nevertheless, the paper shows that the effects also arise in archaea (where the release factors are different) as well as in the UTR downstream of the stop codon. Therefore the main conclusion of the paper is that the variation in stop codon frequencies in bacteria is not due to release factors at all, and I find this quite convincing. Unfortunately however, the paper is not very clear what the alternative explanation is. To me, the most likely explanation is that there is some context dependent mutational process, so that the GC content is not sufficient to predict the triplet frequencies, and triplets with the same GC content would not necessarily have the same frequency in absence of selection. This could be assessed by measuring relative frequencies of TAA, TGA and TAG in UTRs out of frame of the stop codon. Maybe this has already been done in Figs 1b & 1c? It is not quite clear to me what exactly is included in 1b and 1c, and whether this already makes the comparison of in-frame and out-of-frame triplet frequencies.

4. At line 255 there is an analysis of the relationship between stop codon usage and the GC content of the 3'UTR of individual genes. It should firstly be checked whether there is significant variation among genes. Can we reject the hypothesis that the GC content is the same for all genes in one genome? If there is significant variation of GC content among genes in one genome, what causes this variation? If the effect arises from context-dependent mutation, as suggested above, then mutational effects will have to vary systematically in different places in the same genome.

5. The paragraph beginning at line 413 discusses which codon is "most enriched". I would suggest changing this to "most frequent". The word enriched seems to imply that the frequency is increased relative to some prior expectation, but there is no prior expectation in this paragraph. I think here what is meant is simply which codon is most frequent.

6. At line 523 it is stated that there is no evidence that tRNA abundance change precedes codon usage adaptation. This is trying to say that tRNAs follow codon frequencies and not the other way. But there are several papers discussing codon frequency evolution that show that there are alternative stable states of codon usage and tRNA gene copy numbers [1-3]. This means the tRNA influence the codons as well. The interaction goes both ways.

[1] Higgs PG, Ran W (2008) Coevolution of codon usage and tRNA genes leads to multiple stable states of biased codon usage. *Mol. Biol. Evol.* 25: 2279-2291.

[2] Ran W, Higgs PG (2010) The influence of anticodon-codon interactions and modified bases on codon usage in bacteria. *Mol. Biol. Evol*. 27: 2129-2140.

[3] Shields DC (1990) Switches in Species-Specific Codon Preferences: The Influence of Mutation Biases. J Mol Evol (1990) 31:71-80
